# Superior training efficacy of beginning movement load training for the baseball throwers

**DOI:** 10.1186/s13102-021-00357-2

**Published:** 2021-10-13

**Authors:** Wen-Yi Chou, Jih-Yang Ko, Shu-Fang Chen, Chia-Feng Wu, Kuan-Ting Wu, Shun-Wun Jhan

**Affiliations:** 1 Department of Orthopaedic Surgery, Kaohsiung Chang Gung Memorial Hospital and Chang Gung University College of Medicine, 123 Ta Pei Road, Niao Sung Dist., Kaohsiung City, Taiwan; 2 Center of Comprehensive Sports Medicine, Chang Gung Memorial Hospital, Taoyuan City, Taiwan; 3 Department of Leisure and Sport Management, Cheng Shiu University, Kaohsiung City, Taiwan; 4 Department of Neurology, Kaohsiung Chang Gung Memorial Hospital and Chang Gung University College of Medicine, Kaohsiung City, Taiwan

**Keywords:** Beginning movement load training, Baseball, Shoulder, Thrower’s ten

## Abstract

**Background:**

Superior shoulder motion with rotator cuff activation are essential for the performance of the throwing athletes. The present study compared the novel beginning movement load training (BMLT) and popular throwers ten program regarding the training efficacy of baseball throwers. We hypothesized that the BMLT contributed the superior training efficacy than popular throwers ten program.

**Methods:**

Forty adult baseball players were randomized into study group and control group equally. In study group, the cyclic shoulder motion was repeatedly operated 3 days in a week and lasted for 6 weeks using three different BMLT training machines. As for control group, three popular cyclic training in the throwers ten program were adopted for the shoulder trainings as the same protocol in study group. The evaluations before and after training included the static range of motion (ROM), the maximal voluntary isometric contraction (MVICs) of the target muscle (pectoralis major, middle deltoid and supraspinatus) and throwing velocity.

**Result:**

After 6-week course, study group had significant wider static ROM in saggital adduction (*p* = 0.002), coronal internal rotation (*p* = 0.018) and external rotation (*p* = 0.044) than in control group. The maximal voluntary isometric contraction (MVIC) ratio of middle deltoid/supraspinatus was significant lower in study group (Study:Control = 1.14 ± 0.76:3.56 ± 5.57, *p* = 0.049) which indicated the enhanced supraspinatus maximal contraction in the study group after training. In addition, the study group had significant improvement in throwing speed (117 ± 10 vs. 109 ± 10 km/h, *p* = 0.040).

**Conclusion:**

The BMLT contributed the superiority in range of motion, recruitment of supraspinatus and throwing velocity than the popular thrower’s ten program. It could be a favourable training for the overhead activity.

## Introduction

Overhead motion is a very common activity in daily life. In addition, many sports involve overhead motion, such as baseball, javelin, tennis, badminton. Throwing motion involves giving velocity and direction to a specific target, and is accomplished by coordination of the legs, trunk, and arms, well-known as the concept of a kinetic chain [1]. The maximum pitch counts in a game ranged from 130 to 172 and the overhead pitching counts is proportion to the injury and dampen the performance [2]. The throwing motion is generally divided into 5 phase [3] and many studies have revealed the important role of the cocking phase in the whole throwing motion, as it is related to the shoulder injury and performance [4–7]. It is believed that most throwing injuries occur in the cocking phase which contains the maximal external rotation of the shoulder and the subacromial impingement related supraspinatus tendon partial-thickness tears or even full-thickness tears might happen in this stage that has been reported to be the common sources of pain that limit the ability to throw [8, 9]. Regardless of treatment after injury, many exercise modalities have been proposed to prevent injury or enhance the performance of the rotator cuff, especially the supraspinatus, including shoulder external rotation training using an elastic band or light-weight dumbbell, and Thrower’s ten program [3, 10]. Concerning training that mimics the specific cocking phase without the potential complications of open kinetic chain training, a novel training concept with special equipment, beginning movement load training, is being introduced here.

**Fig. 1:**
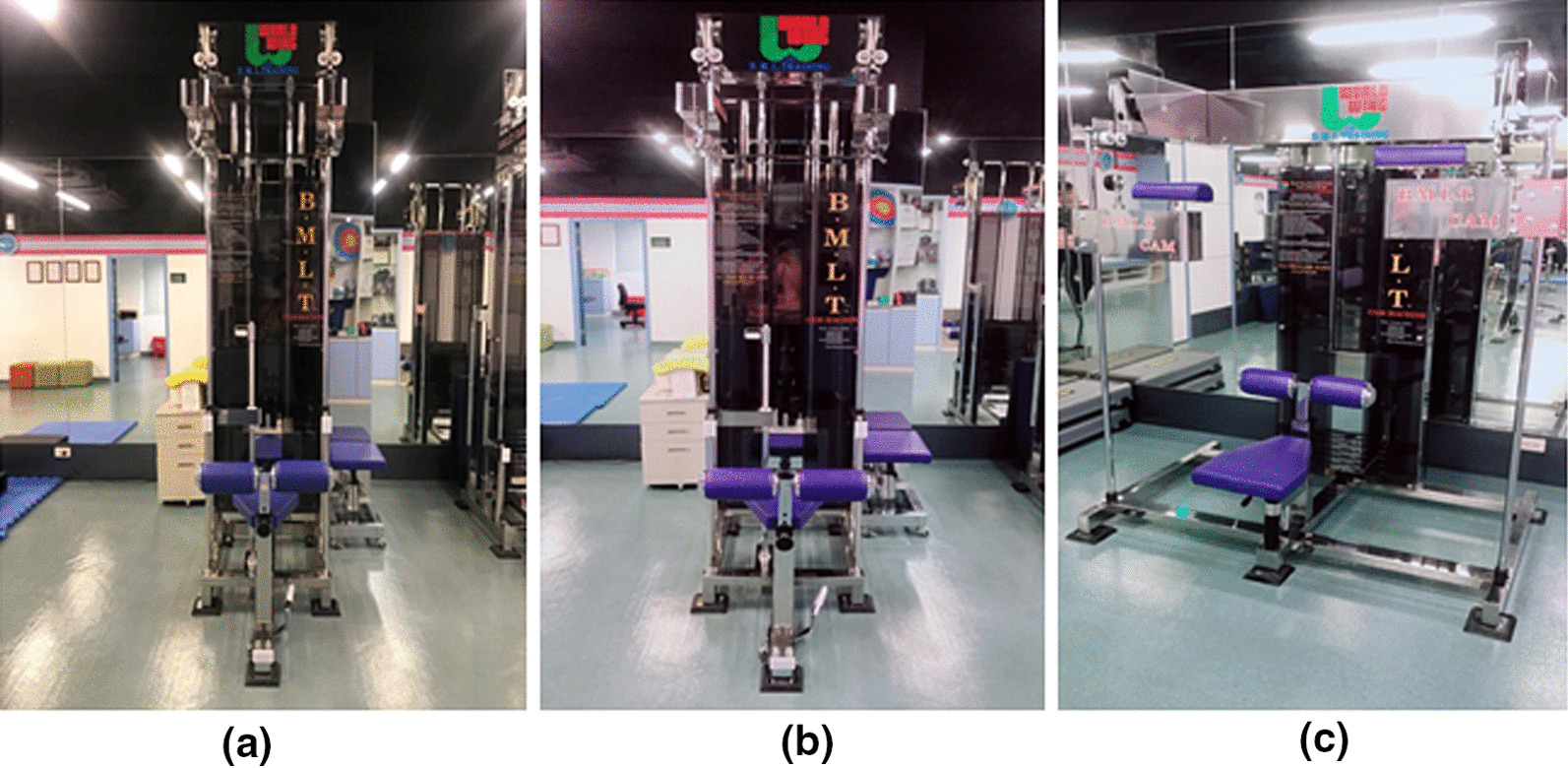
**a** Scapula 2000, **b** Scapula 1000, **c** Clavicle 2000

**Fig. 2:**
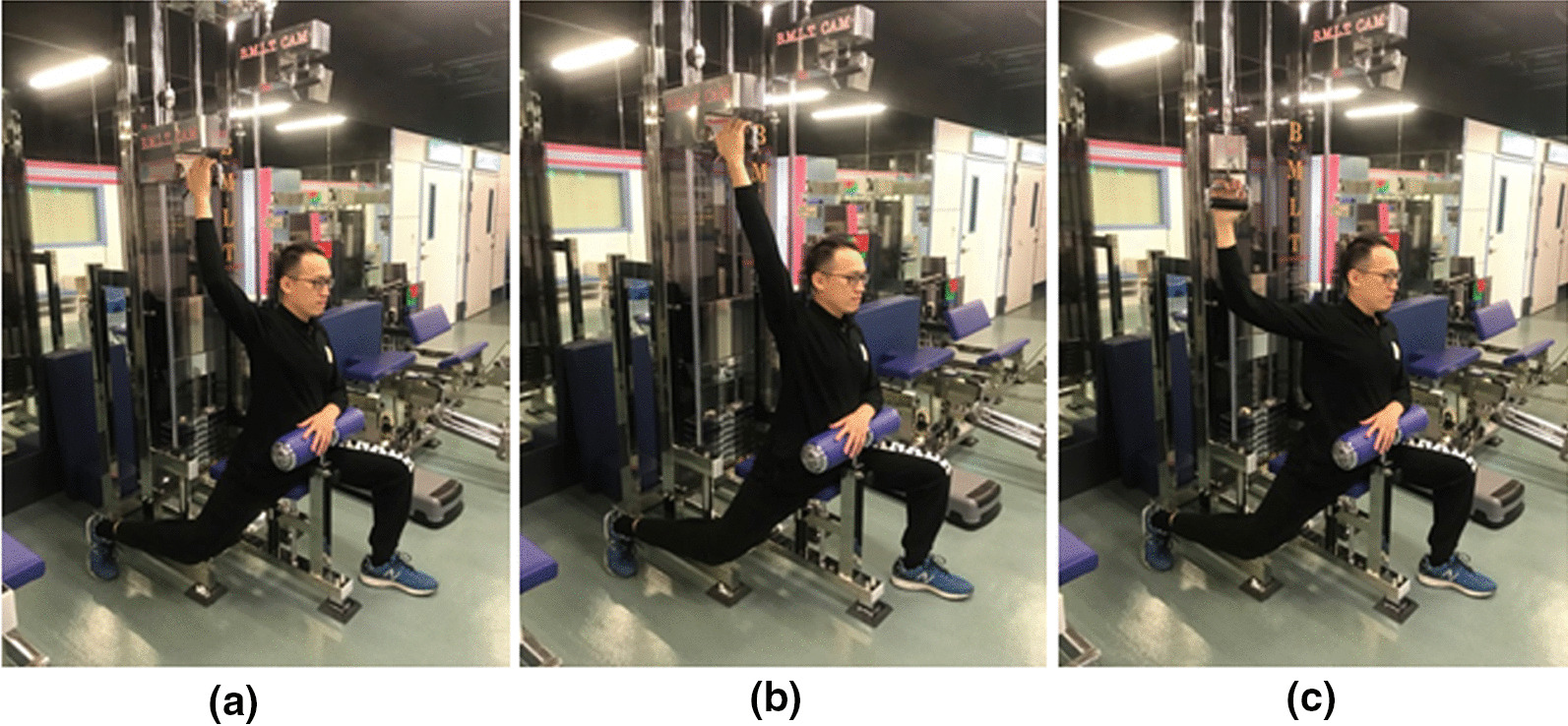
Scapula 2000. **a** Hand on handle with semi-full stretch, relaxation, **b** hand on handle with full lengthening, **c** shoulder horizontal adduction with muscle contraction

**Fig. 3:**
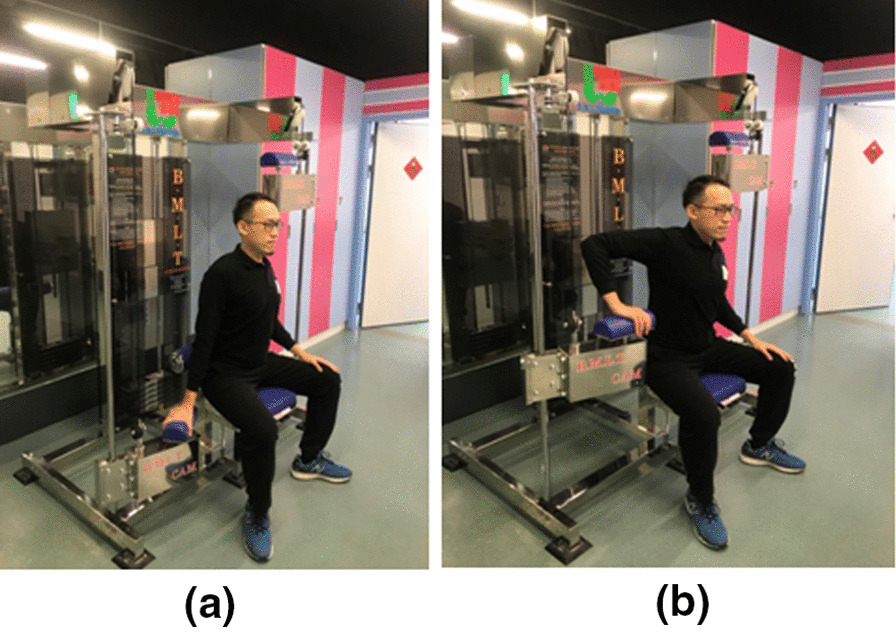
Clavicle 2000. **a** Hand on handle with shoulder 0-degree abduction; **b** hand on handle with shoulder 90-degree abduction and internal rotation

Beginning movement load training (BMLT) and related training equipment were developed by Dr. Koyama in 1994. In contrast to ordinary open kinetic chain training, in which muscle contraction occurs at the start of an exercise, the mainstay of BMLT is relaxing the muscle at the beginning of an exercise, followed by lengthening and contraction. This cyclic motion prevents the co-contraction of agonist and antagonist muscles [11]. Another important characteristic of BMLT is rotation on a long axis at extension and flexion of the extremities, which is defined as the “dodge movement” [12]. With the dodge movement, a short-term relaxation interval emerges before a shortening contraction of an agonist muscle, preventing co-contraction [12]. BMLT employs a specific device composed of a cam-crank mechanism, which permits synchronization of open-closed chain motions in the horizontal direction, in addition to up-down motions in the vertical line, thereby increasing the joint range of motion combined with torsional motion. Using these machines, upper extremity training involves reciprocating motions in the vertical direction and rotation on the horizontal plane; moreover, horizontal extension, flexion, outward/inward rotation, external/internal rotation of the shoulder, extension, and flexion of the elbow, and pronation/supination of the forearm are executed simultaneously (Figs. 1, 2, 3). Compared to the published training modalities [13, 14], like medicine ball, rubber tubing, dumbbells, which are the open chain exercise, this specific training modality simulates common overhead activities, such as the cocking phase of the overhead throwing, which combine the characteristics of open and closed kinetic chains. According to the training rationale and activity, we assert that BMLT is a favorable training modality for the shoulder in light of its complicated muscle activation. This study aimed to compare the differences and outcomes of BMLT and the conventional Thrower’s ten program training for the cocking phase motion (abduction with maximal external rotation) of the baseball throwers. We hypothesized that the BMLT contributed the superior training outcomes, including range of motion, cuff recruitment and velocity, than popular throwers ten program.

## Materials and methods

### Participants

This prospective comparative randomized study was approved by the Institutional Review Board (IRB) of Chang Gung Medical Foundation (IRB Study No. 201600644B0). Forty adult, asymptomatic and active amateur baseball players were enrolled in this study. These athletes were randomized in equal numbers into the study group and control group. Players with musculoskeletal disorders in the last 3 months were excluded due to safety concerns. All the evaluators and training were performed in sports performance training center of Kaohsiung Chang Gung Memorial hospital. All participants read and signed an informed consent form approved by the Institutional Review Board of our hospital.

### Evaluations

Pre- and post-training evaluation of the throwing shoulders included measurement of static range of motion (ROM), dynamic surface electromyography of pectoralis major, middle deltoid and supraspinatus, and throwing velocity. All these parameters were recorded before and after 6-week training.

#### Static range of motion

The shoulder range of motion related to throwing activity was recorded before and six weeks after the training. The goniometer was adopted to record shoulder ROM included saggital flexion/extension, coronal abduction/adduction, and external/internal 90-degree coronal abduction. All the measurements were conducted by one sports-specialized physical therapist that had involved in the study for more than 3 years.

#### Throwing velocity

Following adequate and personal habitual dynamic stretch warm-up (reaching at least 60% of the maximum heart rate), the players were asked to throw five pitches with their greatest effort from the flat ground to a target net 18 m away that simulate the distance from the pitcher plate to the catcher, and the highest pitch velocity was recorded. Throwing velocity was measured using speed radar gun (Stalker sport 2 radar gun, Stalker, USA).

#### Dynamic surface electromyography

Surface electromyography (sEMG) was adopted to assess muscle recruitment and outcomes under different training modalities. The maximal voluntary isometric contractions (MVICs, Newton meter, Nm) of the affected muscles were investigated by performing sequential motion mimicking the cocking phase on the different training machines. The muscles that were investigated that contribute to the cocking phase were the pectoralis major, middle deltoid and supraspinatus. To minimize cross-interference in the myoelectrical signal, the investigation was limited to these 3 muscles simultaneously. Muscle activity was measured using surface electromyography (DTS Belt Receiver/Retransmitter, DTS EMG Probe, Software: MR3, Noraxon, USA). The EMG signal using a standard surface sensor by the computer-based EMG system was recorded as the amplitude. The system has an active electrode that locating the amplifier allows artifacts to be canceled and transferring the signal. The limiting factors, such as noise ratio, sEMG interference signal, and artifacts, in the EMG technique with the spectral variables signaling did not observe in this study. Skin preparation of the affected shoulder was performed prior to sEMG sensor tagging. The pectoralis major was located medial to the axillary fold while the athlete medially rotated the arm against resistance. The electrodes were placed horizontally on the chest wall over the muscle approximately 6 cm below the clavicle. The electrodes for the middle deltoid were placed on the lateral aspect of the upper arm and 3 cm below the acromion, parallel to the muscle fibers. The myoelectrode signal of the supraspinatus was detected by placing of the electrode just above the spine of the scapula and 2 cm from its medial border. Players in the study group were asked to perform shoulder 90-degree coronal abduction with external rotation from 0 degrees to maximum horizontal adduction on the BMLT machine (SCA. BACKS 4D-2000, Worldwing, Tottori, Japan) under a 5 kg weight load. The motion simulated the shoulder motion in the cocking phase. Players in the control group were also asked to perform the same motion, but on a commercialized Cable machine (Dual adjustable pully L370, BH, Spain).

### Training in the study group

In the study group, players were asked to discontinue all upper extremity training except BMLT using the Scapula 2000, Scapula 1000 and Clavicle 2000 machines (Figs. 1, 2, 3). It also emphasized concomitant scapular motion from the upward rotation to scapular retraction. The training sequence of Scapula 2000 started from the position of Fig. 1a–c on a single shoulder. The Scapula 1000 (Fig. 3b) employed the same sequence as the Scapula 2000, with the exception that both upper extremities operated simultaneously. The training on Clavicle 2000 stared from the position of Fig. 2a followed by Fig. 2b. All the sequences emphasized the relaxation in the starting position followed by lengthening and contraction of shoulder. On each machine, the cyclic motion was repeated 15 times per session; the player completed 5 sessions on each machine per day, 3 days a week. BMLT lasted for 6 weeks: it started with a 0 kg load on each machine in the first week, which was increased to 5 kg in the 2nd week, followed by increases of 5 kg per week until reaching 25 kg in the 6th week. The total weight-load during training was 10,125 kg over the 6-week course.

### Training in the control group

Conventional training was carried out using 3 popular cyclic motions, as adopted in the Thrower’s ten program [14] using a Cable machine, which included shoulder diagonal pattern flexion/extension, shoulder abduction/scaption, and external/internal rotation at a 90-degree coronal abduction. Each cyclic motion was repeated 15 times in one session, with 5 sessions for each cyclic motion being completed in 1 day, 3 days a week. All the motions operated in full motion of the affected shoulder and the training finished within 90 min in a day. Conventional training also lasted for 6 weeks: it started with a 0 kg load for each motion in the first week, then the weight was increased to 5 kg in the 2nd week, followed by increases of 5 kg per week, reaching 25 kg in the 6th week. The total weight-load over the 6-week training period was 10,125 kg that was the same as in the study group.

### Statistical analysis

Statistical analysis was performed using SPSS for Windows (Statistical Package for the Social Sciences, version 25.0; SSPS Inc., Pompano Beach, FL, USA). The Wilcoxon test was adopted to analyze the pre-training and post-training conditions, and comparison of the study group with the control group was performed using the Mann–Whitney U test. A value of *p* < 0.05 was accepted as indicative of statistical significance.

## Results

Forty active college baseball players were recruited to participate in this prospective randomized comparative study. Twenty players were assigned to the study group, and twenty to the control group. No significant differences in the pre-training demographic characteristics were observed between groups (Table 1).

**Table 1:** Athlete demographic characteristics

	Study group	Control group	Total	*p* value
Number of athletes	20	20	40	1.0
Dominant hand (R/L)	16/4	18/2	34/6	0.661
Ave. age (years)				
Mean ± SD	22.2 ± 3.30	21.1 ± 1.70	21.6 ± 2.63	0.179
(Range)	(20–33)	(20–26)	(20–33)	
Gender (male/female)	20/0	20/0	40	1.0
Position				
1B	1	3	4	0.851
2B	2	1	3	
3B	2	0	2	
IF	3	4	7	
OF	3	3	6	
Pitcher	8	8	16	
Catcher	1	1	2	

*1B* first baseman, *2B* second baseman, *3B* third baseman, *IF* Infielder, *OF* Outfielder

### Static range of motion

No difference in the static ROM of the shoulder before training was observed between groups. After 6 weeks training, the study group revealed significant increases in saggital extension (*p* = 0.007), coronal adduction (*p* = 0.002) and 90-degree coronal abduction with external (*p* < 0.001)/internal rotation (*p* < 0.001) as compared with the pre-training static ROM, while significant improvements were observed incoronal adduction (*p* = 0.042) and 90-degree coronal abduction with internal rotation (*p* = 0.044, Table 2) in the control group. The study group demonstrated a significantly greater ROM in saggital extension (*p* = 0.012) and coronal internal rotation (*p* = 0.018)/external rotation (*p* = 0.044) than the control group. The results of the present study showed that BMLT resulted in a greater static ROM than conventional training in shoulder saggital extension and external/internal rotation.

**Table 2:** Comparative outcomes of shoulder static range of motion and throwing speed

	Study group	Control group	*p* value^a^	Effect size
(1) Flexion (°)				
Pre-training	179 ± 5 (160–180)	179 ± 2 (170–180)	0.392	0.26
Post-training	180 ± 0 (180)	180 ± 0 (180)	0.357	
* p* value^b^	0.234	0.186		
Effect size	0.20	0.50		
(2) Extension (°)				
Pre-training	64 ± 10 (50–95)	61 ± 14 (35–100)	0.176	0.25
Post-training	70 ± 9 (55–90)	62 ± 10 (40–70)	0.012	
* p* value^b^	0.007	0.708		
Effect size	0.78	0.08		
(3) Abduction (°)				
Pre-training	179 ± 7 (150–180)	179 ± 5 (160–180)	0.368	0.16
Post-training	180 ± 0 (180)	180 ± 1 (175–180)	0.343	
* p* value^b^	0.330	0.287		
Effect size	0.14	0.20		
(4) Adduction (°)				
Pre-training	42 ± 11 (30–70)	44 ± 10 (30–60)	0.893	0.19
Post-training	49 ± 11 (35–75)	52 ± 13 (30–85)	0.878	
* p* value^b^	0.002	0.042		
Effect size	0.81	0.52		
(5) Internal rotation				
Pre-training	70 ± 15 (50–100)	68 ± 16 (40–110)	0.477	0.13
Post-training	86 ± 16 (50–115)	75 ± 14 (50–100)	0.018	
* p* value^b^	< 0.001	0.044		
Effect size	1.06	0.46		
(6) External rotation				
Pre-training	115 ± 16 (80–140)	113 ± 20 (40–140)	0.393	0.11
Post-training	120 ± 16 (85–145)	111 ± 12 (75–125)	0.044	
* p* value^b^	< 0.001	0.663		
Effect size	1.56	0.10		
(7) Speed (KPH)				
Pre-training	114 ± 9 (98–128)	108 ± 9 (90–125)	0.105	0.67
Post-training	117 ± 10 (100–132)	109 ± 10 (94–130)	0.040	
* p* value^b^	< 0.001	0.224		
Effect size	1.09	0.16		

^a^*p* value: comparison between Study group and Control group was analyzed using Bayesian inference statistics

^b^*p* value: comparison between the pre-training and post-training conditions was analyzed using Bayesian inference statistics

### Dynamic surface electromyography

The maximal voluntary isometric contractions (MVICs) of the pectoralis major, middle deltoid and supraspinatus were recorded for analysis of the differences in the muscle recruitment pattern and outcomes following the different training modalities.

**Fig. 4:**
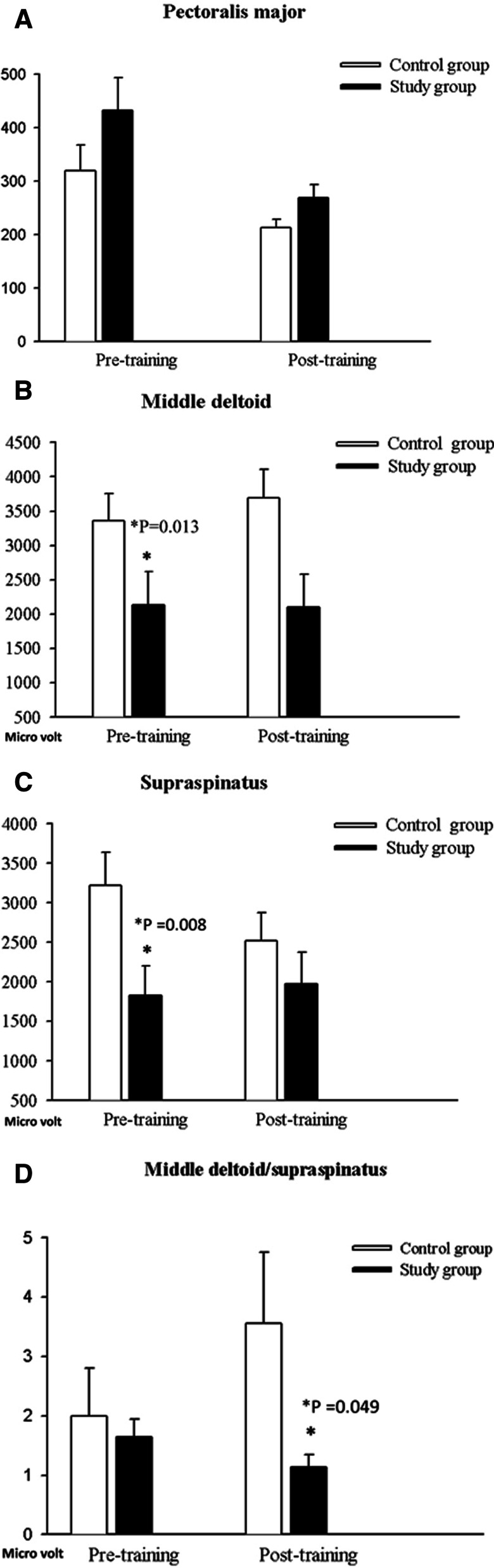
Maximal voluntary isometric contraction **a** pectroralic major, **b** middle deltoid, **c** supraspinatus, **d** middle deltoid/supraspinatus

The MVIC in pectoralis major were no significantly differences in both groups before and after the training (Fig. 4A). The MVIC of middle deltoid and supraspinatus were significantly higher in the control group before training and became statistically insignificance after training (Fig. 4B, C). In addition, the ratio of the middle deltoid versus the supraspinatus became significantly higher in control group after six weeks training (control group vs. study group = 3.56 ± 5.57 vs. 1.14 ± 0.76, *p* = 0.049, Fig. 4D) which indicated that BMLT offered the superior supraspinatus training efficacy than conventional training regarding the synchronized motion of middle deltoid and supraspinatus in cocking motion.

### Throwing velocity

In the analysis of throwing performance, throwing speed is one of the parameters that may reflect the outcome after training. In the present study, the pre-training throwing speed was not significantly different between groups (*p* = 0.105); however, differences were observed after 6 weeks of training. The study group exhibited significant improvement in pitching velocity as compared with the control group (117 ± 10 vs. 109 ± 10 km/h, *p* = 0.040).

## Discussion

To the best of our knowledge, this was the first comparative study to analyze the training outcomes of BMLT for the throwers’ shoulder than popular thrower’s ten program. The principal findings of the present study were that this BMLT yielded a greater range of motion, supraspinatus recruitment in the shoulder cocking motion, and improved the throwing velocity for the baseball players. Hence, the results implied that BMLT is an applicable training alternative for the baseball throwers or overhead activities.

The results of the present study showed that BMLT produced a greater static ROM than conventional training in shoulder saggital extension and external/internal rotation, which is an effect of the most critical feature of BMLT, the three-dimensional training plane. This involves greater scapular movement in the cyclic motion from shoulder elevation to horizontal abduction. In the Thrower’s ten program, most of the resistance or muscular training is carried out on a single plane of motion. Combining the reciprocal “dodge movement” on a stable-handled platform, the characteristics of open and closed kinetic chain exercise are merged, and BMLT increases safety and results in less training-related delayed-onset muscle soreness. Previous studies have demonstrated enhancement of training actions and functional activities by BMLT exercise with a greater range of motion [11, 15]. The results of the present study also revealed that a greater range of motion was obtained using this novel weight training modality. Glenohumeral internal rotation deficit has been related to the throwing injury [16], and the BMLT could serve as a solution owing to the significant improvement of internal rotation for the throwing shoulder.

Synchronization of the middle deltoid and supraspinatus is believed to be critical during shoulder abduction [17, 18] especially in cocking phase. In general, the deltoid muscle plays a major role in shoulder abduction, rather than the supraspinatus. However, the supraspinatus lesion is a much more common injury than the deltoid and the conventional training is difficult in changing rotator cuff activation [19]. In the present study, BMLT resulted in the superior training efficacy of the supraspinatus on the same shoulder loading, which serves as a substantial contribution to the range of motion and overhead performance (Fig. 4). The superior supraspinatus contractions were believed to be positive in improving rotation range of motion and performance in the overhead activity.

The middle deltoid contributes most to movement, while the shoulder abduct in internal rotation and abducts horizontally with external rotation [20–22]. The co-operative mechanism contributes most greatly in the cocking phase [20–22]. The supraspinatus works synchronously with the middle deltoid for shoulder abduction [23]. It has been reported that the supraspinatus is the common injured tendon in the throwing athletes, in particular, partial-thickness tears [8, 9]. Most of the strategies employed for the prevention of supraspinatus injury aim to enhance training or conditioning [24], introduce a proper throwing mechanism [25, 26], and specify an adequate throwing interval program with appropriate rest. As compared with conventional training, the contribution of the supraspinatus increased after 6 weeks of training which indicated that BMLT improved the supraspinatus contribution in the cocking motion over that resulting from conventional training. From the aspects of injury prevention and performance promotion, we postulated that BMLT is an alternative training to provide better supraspinatus training effect for the throwers. Meanwhile, the improvement of throwing velocity were observed in this study although the velocity improvement is multifactorial [27, 28]. Factors related to the velocity gain include weighted/resistance training [29] correction of throwing mechanics, and increased shoulder external rotation [26]. In the present study, we observed that BMLT increased shoulder rotational range of motion and combined the supraspinatus training efficacy that contributed to the throwing speed improvement.

Limitations of the present study exist. First, the infraspinatus muscle, which is involved in shoulder external rotation, was not analyzed in the present study owing to the avoidance of EMG signal interference. Second, EMG analysis should also address muscles that facilitate scapular motion, such as the trapezius, rhomboids, levator scapulae and serratus anterior. Third, a longer training interval and recruitment of a greater number of athletes are required to demonstrate and corroborate the training outcomes. Forth, due to the study group being limited to the amateur level, which involved baseball training for at least 6 years, the identical training efficacy in different level athletes was uncertain.

## Conclusions

The BMLT contributed the superiority in range of motion, recruitment of supraspinatus and throwing velocity than the popular thrower’s ten program. We would like to conclude that BMLT is favourable alternative training modality for the baseball throwers.

## Data Availability

All the data that support the results can be found in the manuscript.

## Abbreviations

BMLT: Beginning movement load training
MVICs: Maximal voluntary isometric contractions
ROM: Range of motion
sEMG: Surface electromyography
